# Thrombospondin 1 (THBS1) Promotes Follicular Angiogenesis, Luteinization, and Ovulation in Primates

**DOI:** 10.3389/fendo.2019.00727

**Published:** 2019-11-07

**Authors:** Hannah R. Bender, Genevieve E. Campbell, Priyanka Aytoda, Allison H. Mathiesen, Diane M. Duffy

**Affiliations:** Department of Physiological Sciences, Eastern Virginia Medical School, Norfolk, VA, United States

**Keywords:** luteinizing hormone, neovascularization, ovarian follicle, ovary, oocyte, macaque, granulosa cell, endothelial cell

## Abstract

Angiogenesis is essential to both ovulation and the formation of the corpus luteum. The thrombospondin (THBS) family of glycoproteins plays diverse roles in regulation of angiogenesis, but the role of these vascular regulators in ovulation and luteinization remain to be elucidated. Using the cynomolgus macaque as a model for human ovulation, we demonstrated that levels of THBS1 mRNA and protein in preovulatory follicle granulosa cells increased after the ovulatory gonadotropin surge, with peak levels just before the expected time of ovulation. THBS1 treatment of monkey ovarian microvascular endothelial cells *in vitro* stimulated migration, proliferation, and capillary sprout formation, consistent with a pro-angiogenic action of THBS1. Injection of an anti-THBS1 antibody into monkey preovulatory follicles reduced rates of follicle rupture and oocyte release in response to an ovulatory gonadotropin stimulus when compared with control IgG-injected follicles. Interestingly, two of three oocytes from anti-THBS1 antibody injected follicles were germinal vesicle intact, indicating that meiosis failed to resume as anticipated. Follicles injected with anti-THBS1 antibody also showed reduced granulosa cell layer expansion, endothelial cell invasion, and capillary formation when compared to control IgG-injected follicles. Overall, these findings support a critical role for THBS1 in follicular angiogenesis, with implications for both successful ovulation and corpus luteum formation.

## Introduction

Angiogenesis is critical for successful ovulation and formation of the corpus luteum (1). New blood vessels form within the dominant follicle in response to the ovulatory gonadotropin surge (2). In primate follicles, capillaries invade the previously-avascular granulosa cell layer before follicle rupture (3). Blockade of key vascular growth factor pathways can prevent both ovulation and luteal formation (1) highlighting the close connection between angiogenesis and ovarian function.

Thrombospondins are a family of vascular regulators, each containing multiple structure/function domains (4). Because of these multiple domains, a molecule of thrombospondin can simultaneously interact with cell surface receptors, extracellular matrix components, matrix remodeling proteases, integrins, and growth factors (4, 5). Thrombospondins have been reported to be both pro-angiogenic and anti-angiogenic. For example, thrombospondin 1(THBS1) most often acts as an anti-angiogenic factor when THBS1's type 1 repeats interact with CD36 receptors (6). In contrast, pro-angiogenic activity of THBS1 has been reported when the N terminal heparin-binding domain interacts with LRP1 receptors (7, 8).

In the ovary, thrombospondins have been examined in growing follicles, where high THBS1 and THBS2 were associated with decreased vascularity and granulosa cell proliferation but increased granulosa cell apoptosis, perhaps contributing to the processes of follicle development and follicle selection (9–12). Expression of THBS1 and THBS2 decline in both granulosa and theca cells of growing antral follicles as follicle size increases; large preovulatory follicles have low levels of thrombospondins in all follicular cell types (9, 11, 13–15). Very limited information on thrombospondin expression after the LH surge suggests that THBS1 and THBS2 levels may in granulosa and theca cells increase transiently after the LH surge (14). THBS1 and THBS2 expression in the young bovine corpus luteum is highest in vascular endothelial cells and vascular smooth muscle (15), highlighting the role of thrombospondins as vascular regulators.

Ovarian thrombospondin expression and action have been studied in growing follicles up to the antral stage and during the process of luteal regression. However, little is known about thrombospondin expression and action during ovulation and transformation of the follicle into the young corpus luteum. The present studies were conducted to examine the expression of thrombospondin family members in the ovulatory follicle of a primate, the cynomolgus macaque. We demonstrate that THBS1 is an LH-stimulated, pro-angiogenic factor during the transformation of the dominant follicle into the young corpus luteum. *In vivo* and *in vitro* studies confirm that THBS1 is critical to the success of primate ovulation.

## Materials and Methods

### Animals

Whole ovaries and ovarian biopsies were obtained from adult female cynomolgus macaques (Macaca fascicularis) at Eastern Virginia Medical School (Norfolk, VA). All animal protocols were conducted in accordance with the National Institutes of Health's Guide for the Care and Use of Laboratory Animals and were approved by the Eastern Virginia Medical School Animal Care and Use Committee. Animal husbandry was performed as described previously (16). Briefly, adult females (aged 4–8 years) with regular menstrual cycles were routinely observed for menstruation; the first day of menstruation marked day 1 of the cycle. Blood samples were obtained with chemical restraint (ketamine, 5–10 mg/kg body weight) as needed by femoral venipuncture and serum was stored at −20°C. Serum estradiol and progesterone levels were determined using the Immulite 1000 immunoassay system (Siemens Medical Diagnostics Solutions, Rockville, MD). Aseptic surgeries were performed by laparotomy in a dedicated surgical suite under isoflurane anesthesia. Postoperative analgesia was accomplished with buprenorphine and a non-steroidal anti-inflammatory drug (ketoprofen or meloxicam).

### Ovarian Stimulation

An ovarian stimulation model was used to obtain ovaries with multiple ovulatory follicles (17). Beginning within 3 days of initiation of menstruation, monkeys received 90 IU of recombinant human follicle stimulating hormone (FSH; Merck and Co., Inc., Kenilworth, NJ) for 6–8 days, followed by 2–3 days of 90 IU of FSH plus 60 IU of recombinant human LH (Serono Reproductive Biology Institute, Rockland, MA) to stimulate the growth of multiple follicles. Animals also received a GnRH antagonist [30 μg/kg Ganirelix (Merck)] daily to prevent an endogenous ovulatory LH surge. Follicular development was monitored by ultrasonography and rising serum estradiol. During aseptic surgery, aspiration of follicles <4 mm was performed before (0), 12, 24, or 36 h after administration of 1000 IU of recombinant human chorionic gonadotropin (hCG; Serono). To inhibit follicular prostaglandin production during the periovulatory interval, some animals were treated as described above; these animals also received the PTGS2 inhibitor celecoxib (32 mg orally every 12 h; Pfizer, New York, NY) beginning with hCG administration and continuing until surgery (16). This treatment has been previously shown to significantly reduce follicular PGE2 levels (16).

### Controlled Ovulation With Follicle Injection

A model of controlled ovulation with follicle injection model was used to introduce an antibody into the ovulatory follicle (18). Beginning on day 5–8 of the menstrual cycle, animals were monitored for rising serum estradiol to indicate development of a large pre-ovulatory follicle. Animals then received a GnRH antagonist (Acyline, 60 μg/kg; NICHD, Rockville, MD) to prevent endogenous LH surge concomitant with 80 IU of FSH and 60 IU of LH for 2 days to maintain healthy development of the follicle. On the next day, intrafollicular injection of an antibody against THBS1 (R&D Systems, Minneapolis, MN; AF3074; *n* = 4) or control IgG antibody (Abbiotec, San Diego, CA; *n* = 4) was performed during aseptic surgery; an estimated 10 μg of antibody protein was delivered to each follicle at injection (Supplemental Table 1). Immediately post-operatively, 1000 IU hCG (Serono) was administered to initiate ovulatory events. Ovariectomy was performed 48 h after follicle injection and hCG, with ovulation anticipated at about 40 h (19). Ovaries were photographed *in situ* before ovariectomy when possible. In one case (control IgG), the ovulation site was obscured from view *in situ* and was photographed after removal. Data from three of these control IgG ovaries has been previously reported (18); a fourth control IgG-injected ovary was obtained from the same cohort of animals that provided the THBS1 antibody injected ovaries and serves as an additional, contemporary control.

### Tissue Preparation

Monkey granulosa cells and oocytes were pelleted from the follicular aspirates by centrifugation at 250 X g. The supernatant (follicular fluid) was removed and stored at −80C. After oocyte removal, a granulosa cell-enriched population of the remaining cells was obtained by Percoll gradient centrifugation (16). Granulosa cells were either used immediately for cell culture or were frozen in liquid nitrogen and stored at −80C. Viability of granulosa cell-enriched preparations was assessed by trypan blue exclusion and averaged 80%. Whole ovaries were bisected such that at least two ovulatory follicles <4 mm in diameter were present on each piece. Pieces were fixed in 10% formalin and embedded in paraffin.

### Quantitative PCR (qPCR)

Levels of mRNA for *THBS1, THBS2*, and *THBS4* were assessed by qPCR using a Roche Lightcycler (Roche Diagnostics, Atlanta, GA). Total RNA was obtained from granulosa cells, treated with deoxyribonuclease and reverse transcribed as previously described (20). PCR was performed using the FastStart DNA Master SYBR Green I kit (Roche) following manufacturer's instructions. Primers were designed based on human or monkey sequences and span an intron to prevent undetected amplification of genomic DNA. PCR products were sequenced (Genewiz, South Plainfield, NJ) to confirm amplicon identity (Supplemental Table 2). All data are expressed as the ratio of mRNA of interest to *ACTB* mRNA for each sample, where a value of 1.0 indicates the same number of copies of mRNA of interest and copies of *ACTB* in a given mRNA sample.

### Histology

Whole ovaries were fixed in 10% formalin for 24 h and embedded in paraffin, oriented such that sections included the follicle apex and follicle wall opposite the apex at the maximal follicle diameter in order to ensure optimal view of the follicle apex (18). Ovaries were serially sectioned at 5 μm with each section retained in order. Every fifth section was deparaffinized in xylene baths, rehydrated through a series of ethanol washes, stained with 30% hematoxylin and 60% eosin (Sigma-Aldrich, St. Louis, MO), and coverslipped with Permount mounting medium (ThermoFisher; Suwanee, GA). Stained sections were imaged using an Olympus microscope with either a DP70 or DP74 digital camera system and associated software (Olympus, Melville, NY). Whole follicle images were assembled from multiple microscopic images of a single tissue section using Image Composite Editor (Microsoft Corp., Redmond, WA).

At least two independent observers performed histologic evaluation of sections from each ovary. Evaluation of sections included identification of the oocyte and (if present) condition of the cumulus (tight/expanded) as well as presence/absence of a rupture site. The size of each rupture site was quantified by measuring the width on the section with the largest rupture site, counting the number of 5 μm sections where the rupture site was present, and using these measurements to calculate the area of an oval.

### Western Blot

Granulosa cell lysate preparation and western blot were performed essentially as previously described (3). Briefly, granulosa cell lysates were loaded onto a 3–8% polyacrylamide gradient gel (ThermoFisher). Proteins were transferred to a polyvinylidene fluoride membrane (Immobilon; Millipore, Billerica, MA) and probed using antibodies against THBS1 (0.01 μg/ml; goat; R&D Systems), THBS4 (0.01 μg/ml; goat; R&D Systems), or actin (5 μg/ml; mouse; Millipore) (Supplemental Table 1). Membranes were incubated with AP-conjugated secondary antibodies (1:5000; Applied Biosystems; Invitrogen, Carlsbad, CA) and protein bands visualized with Tropix CDP-Star according to the manufacturer's instructions (Applied Biosystems; Invitrogen). Pixel density of each band was quantified by ImageJ (https://imagej.nih.gov/). Each sample is expressed as pixel density of the THBS band divided by the pixel density of the actin band.

### Monkey Ovarian Microvascular Endothelial Cells (mOMECs)

Proliferating populations of ovarian microvascular endothelial cells (mOMECs) were obtained from monkey ovulatory follicles, characterized, and maintained as previously described (3).

To assess mOMEC proliferation, cells were grown on glass chamber slides (Nunc, ThermoFisher) until 50% confluent. Cells were incubated overnight in basal media (Lonza, ThermoFisher) with 1% fetal bovine serum (Gibco, Gaithersburg, MD). The next day, cells were treated with recombinant human THBS1 (0.01–1 nM; R&D Systems). After 24 h of treatment *in vitro*, cells were fixed in 10% formalin for Ki67 detection and quantification of proliferating cells by immunocytochemistry as described below.

Migration of mOMECs was assessed as previously described (3) using 6-well plate inserts with 8 μm pores (BD) with cells in basal media inside the insert. Media in the well-consisted of basal media with or without the addition of THBS1 (0.001-1 nM; R&D Systems). For some experiments, THBS1 (1 nM) was preincubated for 24 h at 4C with the THBS1 antibody (R&D Systems), with 4-fold molar excess of antibody. Cells were incubated with treatments for 24 h, then cells on the inside of the insert were removed with a cotton swab. The inserts were fixed in 70% ethanol and stained with hematoxylin and eosin. Five areas of each insert were photographed, and the number of migrated cells was counted for each image.

To assess sprout formation, mOMECs adhered to microcarrier beads were embedded in a fibrin matrix as previously described (3). Basal media plus 0.05 U/mL aprotinin with or without THBS1 (0.01–1 nM; R&D Systems) was added on top of matrixes. Representative beads were photographed before adding treatments (Day 0). For each well, four areas were photographed on Day 1 and Day 2 after initiation of cultures. Beads with sprouts that were entirely within the frame were used to generate sprout counts and measurements. For sprout counts, the number of sprouts per bead was determined, and an average number of sprouts/bead was determined for each treatment group. Sprouts were measured from the edge of the bead to the tip of the longest branch; lengths were averaged for each treatment group.

Human umbilical vein endothelial cells [HUVECs; American Type Culture Collection (ATCC), Manassas, VA] were cultured as described for mOMECs and used as a control primary endothelial cell population.

### Immunohistochemistry

Immunostaining was performed using paraffin-embedded ovaries sectioned at 5 μm essentially as previously described (18). Briefly, tissue sections were heated and deparaffinized. Slides to be used for immunodetection of THBS1 or THBS4 were exposed to antigen retrieval using Tris-ethylenediaminetetraacetic acid (EDTA) as previously described (18). When staining for von Willebrand Factor (VWF) or Ki67, antigen retrieval was not performed. All tissues were blocked with 5% non-immune serum in PBS containing 0.1% Triton X-100. Slides were incubated overnight with primary antibody against THBS1 (2.5 μg/ml; R&D Systems), THBS4 (2.9 μg/ml; R&D Systems), VWF (5 μg/ml; Dako, Carpinteria, CA), or Ki67 (0.35 μg/ml; Dako) (Supplemental Table 1) and color-developed using the Vectastain Rabbit ABC kit (Vector Laboratories, Burlingame, CA). Slides were counterstained in hematoxylin, dehydrated, and permanently coverslipped. All images were obtained using an Olympus BX41 microscope fitted with a DP70 digital camera and associated software. Specificity of THBS1 and THBS4 antibody detection were confirmed by preabsorption of the primary antibody with recombinant human peptides (R&D Systems; source) at a molar ratio of 10:1 antigen:antibody at 4C overnight and use of preabsorbed antibody for immunostaining as described above. Omission of the primary antibody served as an additional negative control.

### Assessment of Luteinization and Angiogenesis

Granulosa cell layer thickness was assessed as previously described (18). Briefly, an ovarian section stained with hematoxylin and eosin was selected which included the maximal diameter of the follicle and the rupture site (if rupture occurred) or thinnest portion of the remaining follicle wall (if rupture did not occur). The granulosa cell layer immediately opposite the apex or thinnest portion of the remaining follicle wall was assessed. The distance from granulosa cell basement membrane to antral edge of granulosa cells was measured, with a minimum of eight replicate measurements made for each ovarian tissue.

Assessment of endothelial cell invasion into granulosa cell layer (18) was performed using ovarian tissues immunostained for the endothelial cell protein VWF. Endothelial cell invasion into the granulosa cell layer was assessed using a tissue section which included the maximal diameter of the follicle and the rupture site or thinnest portion of the remaining follicle wall. The granulosa cell layer immediately opposite the apex or thinnest portion of the remaining follicle wall was assessed. The distance from the granulosa cell basement membrane to the VWF^+^ cell closest to the follicle antrum was determined, with at least five replicate measurements made for each tissue section evaluated.

Three dimensional (3D) modeling of capillary sprouting from stromal vessels was performed as previously described (18). Briefly, four adjacent 5 μm ovarian sections were immunostained for VWF as described above. VWF^+^ cells from these adjacent sections were traced into WinSurf software (WinSurf Technology, Hertfordshire, UK) and reconstructed following the manufacturer's instructions.

### Data Analysis

Data were assessed for heterogeneity of variance by Bartlett's tests. Data were log transformed when Bartlett's test yielded *p* < 0.05; log-transformed data were subjected to Bartlett's test to confirm that *p* > 0.05. All data sets were assessed by unpaired *t*-test, paired *t*-test, or ANOVA (without or with repeated measures) as indicated in the text and figure legends. ANOVA was followed by Duncan's multiple range test when *p* < 0.05. Statistics were performed using StatPak version 4.12 software; Northwest Analytical, Portland, OR. Significance was assumed at *p* < 0.05. Data are expressed as mean ± SEM.

## Results

### Ovulatory Follicles Produce Thrombospondins in Response to the Ovulatory Gonadotropin Surge

**Figure 1:**
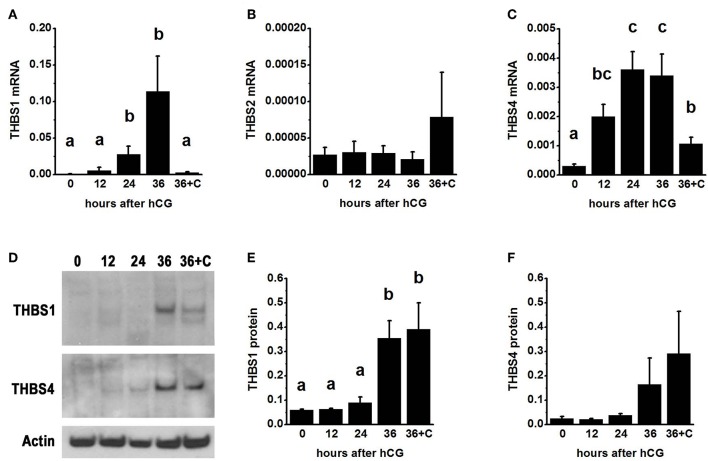
Thrombospondin production by the ovulatory follicle. Granulosa cells were aspirated from monkey ovarian follicles after ovarian stimulation before (0), 12, 24, or 36 h after administration of an ovulatory dose of hCG; additional monkeys received hCG and the PTGS2 inhibitor celecoxib (36+C) 36 h before follicle aspiration. Granulosa cells were assessed by qPCR for mRNA levels of *THBS1* **(A)**, *THBS2* **(B)**, and *THBS4* **(C)**. All *THBS* mRNA levels are expressed relative to *BACT*. Granulosa cell lysates were assessed for THBS1 **(D,E)** and THBS4 **(D,F)** by western blotting and are expressed relative to pan-actin **(D)**. For **(A–C,E,F)**, data are expressed as mean + SEM, *n* = 3–7 samples/treatment. Within each panel, groups with no common letters are different by ANOVA and Duncans *post hoc* test, *p* < 0.05.

To examine thrombospondin synthesis and accumulation in the ovulatory follicle, granulosa cells were obtained from monkeys experiencing ovarian stimulation either before (0 h) or 12–36 h after administration of an ovulatory dose of hCG to span the ovulatory interval in primates. Total granulosa cell RNA was assessed for *THBS1, THBS2*, and *THBS4*. *THBS1* mRNA was low 0–12 h after hCG and was significantly elevated 24–36 h after hCG (Figure 1A). In contrast, *THBS2* mRNA levels were low and unchanging throughout the ovulatory interval (Figure 1B). *THBS4* mRNA was low at 0 h and significantly elevated 12–36 h after hCG (Figure 1C). Overall, the highest mRNA copy numbers were observed for *THBS1*, with lower copy numbers for *THBS4* and very low copy numbers for *THBS2*.

To determine if changes in *THBS1* and *THBS4* mRNA were accompanied by similar changes in thrombospondin proteins, granulosa cell lysates were subjected to western blotting. THBS1 protein was detected as a single band of 160 MW (Figure 1D). Granulosa cell THBS1 was low 0–24 h after hCG, then rose to higher levels at 36 h hCG (Figure 1E). THBS4 was detected as a single band of 130 MW (Figure 1D). There was no significant change in granulosa cell THBS4 protein over the ovulatory interval, though a trend toward increasing THBS4 was noted (Figure 1F).

**Figure 2:**
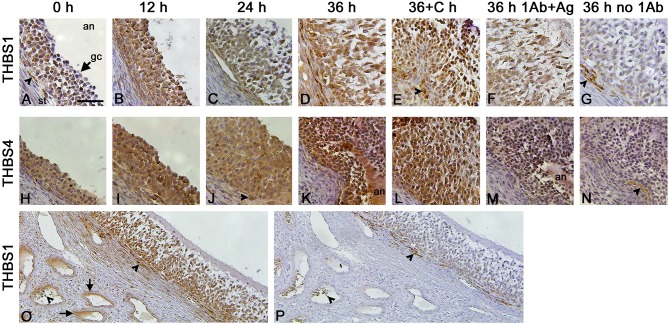
THBS1 and THBS4 immunodetection in monkey ovarian follicles. Immunocytochemical detection (brown) of THBS1 **(A–G)** and THBS4 **(H–N)** was localized to the granulosa cell layer of ovulatory follicles obtained before (0; A,H) and 12 **(B,I)**, 24 **(C,J)**, and 36 **(D,K)** hours (h) after hCG administration as well as 36 h after administration of hCG and celecoxib (36+C h; **E,L**). Images shown are representative of n-3-6 ovaries/treatment. Granulosa cell immunodetection of THBS1 **(F)** and THBS4 **(M)** was reduced after preabsorption of the primary antibody with recombinant human THBS1 or THBS4 and similar to staining observed with no primary antibody **(G,N)**. Nuclei are counterstained blue. All panels are oriented as shown in **(A)**, with stroma (st) in lower left, granulosa cell (gc, arrow) layer central, and follicle antrum (an) in upper right. Arrowheads indicate stromal staining with antibody against THBS1 **(A,E)**, THBS4 **(J)**, and no primary antibody **(G,N)**. Images in **(A–N)** are at the same magnification; bar in **(A)** is 50 μm. Lower magnification images shows immunocytochemical detection of THBS1 **(O)** and no primary antibody **(P)** stained sections of an ovary obtained after ovarian stimulation and 36 h hCG. Staining is apparent in stromal vessels (**O**, arrows). Non-specific staining is indicated near the granulosa cell basement membrane and in vessel lumens in **(O,P)** (arrowheads); bar in **(A)** is 100 μm for images in **(O,P)**.

Granulosa cells were the primary ovarian cell type containing immunodetectable thrombospondin proteins. THBS1 was detected in granulosa cells, with little staining present in the ovarian stroma (Figures 2A–D). Similarly, THBS4 was detected primarily in granulosa cells (Figures 2H–K). Specificity of these primary antibodies was supported by identification of a single band on Western (Figure 1D) and confirmed by reduction in immunostaining following preabsorption of the antigen, which appeared similar to the absence of primary antibody, for THBS1 (Figures 2F,G) and THBS4 (Figures 2M,N). Walls of major stromal vessels also showed evidence of THBS1 immunodetection (Figure 2O). Occasional cells in the stroma underlying the granulosa cell basement membrane also showed staining for THBS1 (Figures 2A,E) and THBS4 (Figure 2J). However, similarly-located cells were stained when the primary antibody was omitted (Figures 2G,N–P), so this stromal staining may not represent specific detection of THBS proteins.

Previous studies demonstrated that follicular levels of PGE2, a key ovulatory mediator, increase 24–36 h after hCG in macaque follicles (16). To determine if prostaglandins mediate the hCG-stimulated increase in granulosa cell thrombospondin mRNA or protein levels, additional monkeys experiencing ovarian stimulation also received the PTGS2 inhibitor celecoxib, which blocks follicular prostaglandin synthesis and reduces follicular PGE2 concentrations to very low levels (16). Celecoxib treatment reduced the increase in *THBS1* and *THBS4* mRNAs in granulosa cells collected 36 h after hCG (Figures 1A,C). However, celecoxib did not alter granulosa THBS1 and THBS4 protein levels when compared to hCG only after 36 h of treatment (Figures 1E,F). Finally, celecoxib treatment did not alter THBS2 mRNA (Figure 1B) or result in an apparent change in THBS1 or THBS4 immunodetection in monkey ovarian follicles (Figures 2E,L).

### THBS1 Regulates Angiogenic Functions *in vitro*

Endothelial cells obtained from monkey ovulatory follicles (mOMECs) were used for *in vitro* studies to explore the role of thrombospondin in angiogenesis of the ovulatory follicle. THBS1 was selected as an agonist for these studies since *THBS1* mRNA was expressed at the high levels in granulosa cells, and THBS1 protein showed significant increase with hCG *in vivo* (Figure 1).

**Figure 3:**
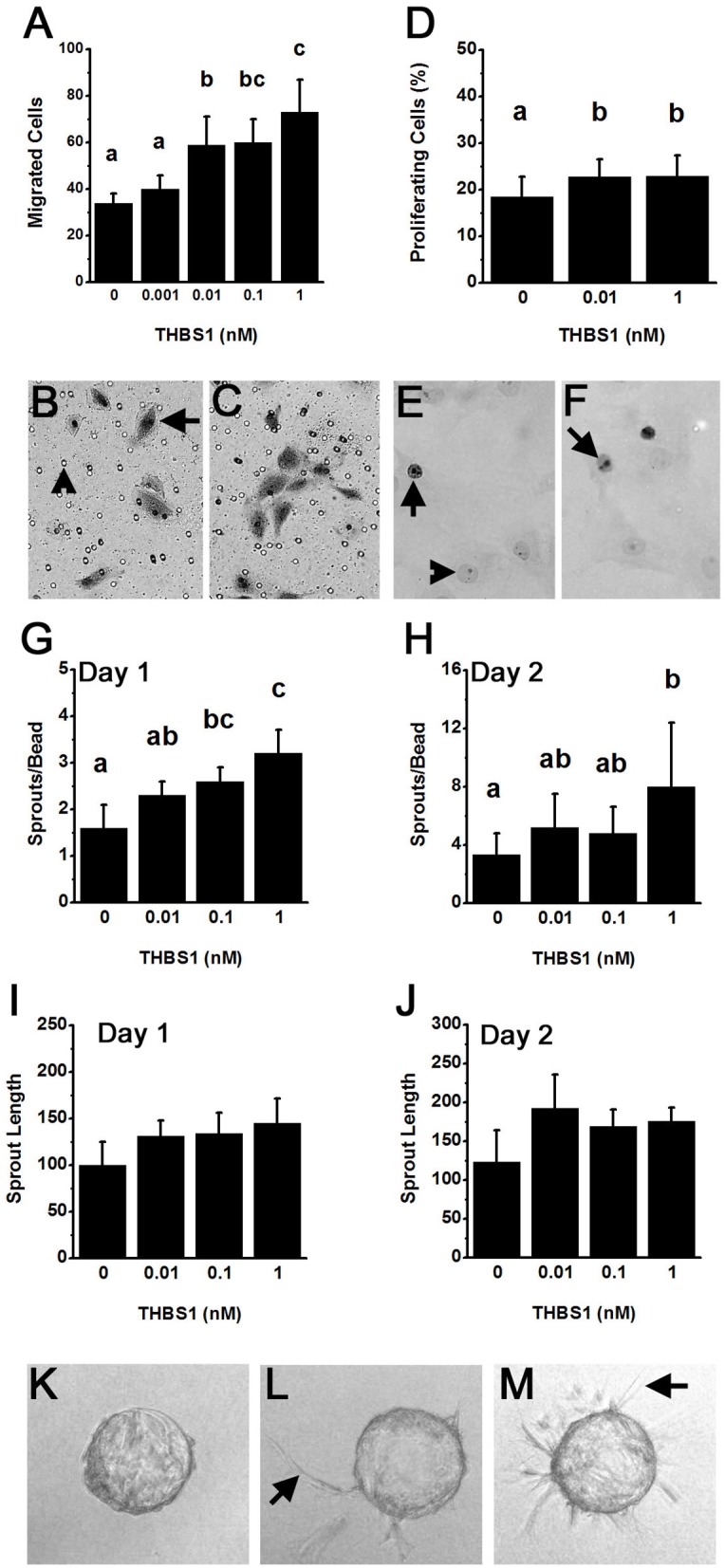
THBS1 is pro-angiogenic *in vitro*. Monkey ovarian microvascular endothelial cells (mOMECs) were treated with human THBS1 protein at concentrations of 0.001–1 nM or no THBS1 (0 nM) as indicated. **(A–C)** Migration was assessed 24 h after plating on a porous membrane and treatment with THBS1. mOMECs (arrow) which migrated through pores (arrowhead) were stained with hematoxylin and eosin, photographed, and counted. Representative membranes from 0 nM **(B)** and 1 nM **(C)** treatment groups are shown. **(D–F)** Proliferation was assessed by Ki67 immunodetection in mOMECs cultured for 24 h with THBS1. Ki67 positive (arrows) and negative (arrowhead) cells are indicated in representative images from mOMECs treated with 0 nM **(E)** and 1 nM **(F)** THBS1. Data are expressed as a percentage of Ki67 positive cells among all cells counted. **(G–M)** Sprout formation in response to THBS1 treatment was determined after 1 day **(G,I)** and 2 days **(H,J,L,M)** *in vitro*. mOMECs coating a polymer bead *in vitro* before THBS1 treatment (Day 0; **K**) shows absence of sprouts. Arrows indicate representative sprouts on Day 2 of treatment with no THBS1 **(L)** and 0.1 nM THBS1 **(M)**. Images were quantified for both the number of sprouts (sprouts/bead; **G,H**) and sprout length in μm **(H,J)**. For **(A,D,G–J)**, data are expressed as mean + SEM, *n* = 3–5 samples/treatment. Within each panel, groups with no common letters are different by ANOVA and Duncans *post hoc* test, *p* < 0.05.

Capillary formation involves endothelial cell migration and proliferation to form a new capillary sprout (21). mOMECs showed increased migration in response to THBS1 (Figures 3A–C). THBS1 increased mOMEC migration in a dose-dependent manner, with 0.01–1 nM concentrations of THBS1 stimulating an increase in migrated cells (Figure 3A). THBS1 also stimulated a small but significant increase in mOMEC proliferation *in vitro* (Figures 3D–F).

An *in vitro* model was used to determine if THBS1 stimulated mOMECs to form new capillary-like endothelial cell sprouts (Figures 3G–M). THBS1 treatment increased the number of sprouts formed in a dose-dependent manner after 1 day in culture when compared to sprouts formed in media without THBS1 (Figure 3G). A similar dose-dependent increases in sprout number was observed after 2 days in culture (Figure 3H). THBS1 treatment did not alter sprout length at any dose after 1 day (Figure 3I) or 2 days *in vitro* (Figure 3J). However, analysis of all sprout length data identified a small but significant increase in sprout length in response to THBS1 treatment when compared with no treatment (ANOVA with two repeated measures, *p* < 0.05).

The ability of THBS1 to stimulate pro-angiogenic actions in mOMECs contrasts with the majority of reports demonstrating a role for THBS1 as an angiogenesis inhibitor (22). For this reason, a common primary endothelial cell population, human umbilical vein endothelial cells (HUVECs), was assessed in our migration assay. There was no difference between the number of migrated HUVECs after 24 h treatment with no THBS1 (14.7 ± 2.7), 0.1 nM THBS1 (10.6 ± 1.1), or 10 nM THBS1 (15.2 ± 2.6) (*p* > 0.05, *n* = 3 independent experiments).

### THBS1 Antibody Reduces Ovulation *in vivo*

**Figure 4:**
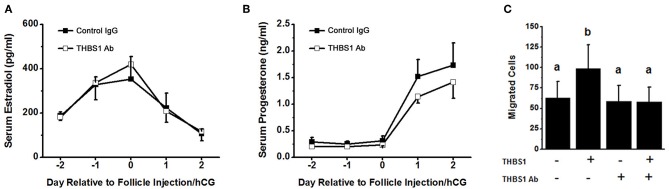
Confirmatory experiments for the intrafollicular antibody injections. Serum levels of estradiol **(A)** and progesterone **(B)** were not different between animals receiving intrafollicular injection of the THBS1 antibody and animals receiving control IgG. For each day, hormone levels were compared by unpaired *t*-test. Data are expressed as mean + SEM, *n* = 4/group. **(C)** THBS1 antibody reduces THBS1 (1 nM)-stimulated mOMEC migration *in vitro*. Within each panel, groups with no common letters are different by ANOVA with 1 repeated measure and Duncans *post hoc* test, *p* < 0.05. Data are expressed as mean + SEM, *n* = 3/group.

To examine the role of THBS1 in primate ovulation, luteinization, and follicular angiogenesis, preovulatory monkey follicles were injected with an antibody against THBS1 or control IgG. An ovulatory dose of hCG was administered immediately after follicle injection. The ovary was removed 48 h later, with ovulation is anticipated at about 40 h after the ovulatory gonadotropin stimulus. Serum estradiol and progesterone were not different between treatment groups before or after follicle injection (Figures 4A,B). To confirm that the THBS1 antibody neutralizes THBS1 bioactivity, THBS1 was preincubated with the THBS1 antibody before use in the mOMEC migration assay. In these experiments, THBS1 (1 nM) increased migration (Figure 4C). Preincubation of THBS1 with the THBS1 antibody reduced migration to levels measured in cells cultured without THBS1. Treatment with the THBS1 antibody alone did not increase or decrease migration compared with untreated cells (Figure 4C).

**Figure 5:**
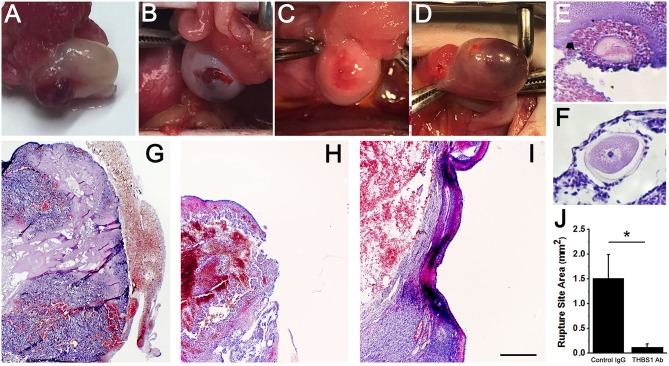
Follicle rupture and oocyte release are compromised after intrafollicular injection with an antibody against THBS1. Ovarian surface at the time of ovary removal after intrafollicular injection of control IgG **(A)** or THBS1 antibody **(B–D)**. Oocytes were located within a THBS1 antibody-injected follicle **(E)** and on the surface of a THBS1 antibody-injected follicle **(F)**. Rupture site after intrafollicular injection of control IgG **(G)**. Rupture sites in follicles injected with the THBS1 antibody were either small **(H)** or absent **(I)**. Tissues shown in **(E–I)** were stained with hematoxylin and eosin. Oocytes **(E,F)** shown at approximately maximal diameter. Images in **(G–I)** are at the same magnification; bar in **(I)** = 0.5 mm. **(J)** Rupture site area after intrafollicular injection with control IgG (IgG) or THBS1 antibody. Groups are different by two-tailed unpaired *t*-test as indicated by *p < 0.05. Data are expressed as mean + SEM, *n* = 4/group.

Control IgG-injected ovaries showed evidence of normal ovulation and luteinization. A protruding and bloody ovulatory stigmata was visible on each control IgG-injected ovary at the time of removal at surgery (Figure 5A). For each control IgG-injected ovary, the presence of a single rupture site was confirmed upon histological examination (Table 1, Figure 5G). Oocytes were not identified within any of the control IgG-injected follicles (Table 1).

**Table 1:** Oocyte retention and follicle rupture.

	**Control IgG**	**THBS1 Ab**
Oocyte retained in ovary	0/4	2/4
Rupture site present	4/4	2/4

Follicle rupture was reduced in THBS1 antibody-injected ovaries (Table 1). Classical ovulatory stigmata were not observed on the surface of any THBS1 antibody-injected ovary (Figures 5B–D). Appearances ranged from small disruptions of the ovarian surface (Figures 5B,C) to formation of an enlarged hemorrhagic, but unruptured, follicle (Figure 5D). Histological examination showed that two of four THBS1 antibody-injected ovaries possessed very small rupture sites (Figure 5H), and two of four showed no breach in the ovarian surface (Figure 5I). Overall, THBS1 antibody-injection reduced the area of the ovulation site when compared with control IgG-injected ovaries (Figure 5J).

Oocytes were not located in any of the control IgG injected ovaries. However, two oocytes were located within unruptured follicles of THBS1 antibody-injected ovaries (Table 1, Figure 5E). One additional oocyte in expanded cumulus was located on the exterior surface of a THBS1 antibody-injected ovary, just outside the rupture site (Figure 5F). Interestingly, two of the three oocytes identified were germinal vesicle (GV) intact (Figure 5F), despite 48 h of exposure to an ovulatory dose of hCG.

### THBS1 Antibody Alters Luteinization and Angiogenesis *in vivo*

**Figure 6:**
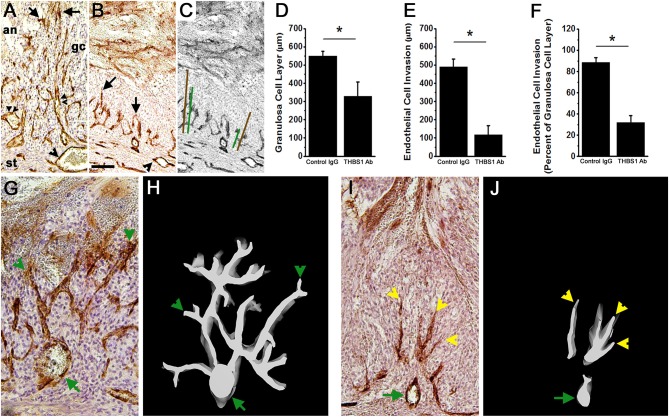
Angiogenesis and luteinization are compromised after intrafollicular injection with an antibody against THBS1. Histological sections of ovaries shown in Figure 5 were immunostained for VWF (brown) to assess angiogenesis; hematoxylin counterstain. **(A,B)** Endothelial cell invasion into the granulosa cell layer of a control IgG-injected follicle **(A)** and THBS1 antibody-injected follicle **(B)** shows VWF+ cells within the granulosa cell (gc) layer. Arrows indicate VWF+ cells which have invaded furthest from the stroma into the granulosa cell layer. Single arrowheads indicate stromal vessels. Double arrowheads indicate capillary luminal spaces with red blood cells in the control-IgG injected follicle only. **(C)** shows representative measurements of granulosa cell layer thickness (brown lines) and endothelial cell invasion (green lines). **(A–C)** are oriented as shown in **(A)**, with stroma (st) at bottom, granulosa cell (gc) layer central, and follicle antrum (an) at top. Images in **(A–C)** are at the same magnification; bar in **(B)** is 100 μm. Granulosa cell layer thickness **(D)**, endothelial cell invasion **(E)**, and the percent of the granulosa cell layer penetrated by endothelial cells **(F)** after intrafollicular injection with control IgG (IgG) or THBS1 antibody. Groups are different by two-tailed unpaired *t*-test as indicated by *p < 0.05. Data are expressed as mean + SEM, *n* = 4/group. **(H,J)** 3D modeling of endothelial cells (white on black background) is shown alongside **(G,I)** representative VWF immunostained ovarian sections after intrafollicular injection with control IgG **(G,H)** or THBS1 antibody **(I,J)**. Green arrows indicate stromal vessels, green arrowheads indicate capillary-like structures that connect to a stromal vessel, and yellow arrowheads indicate endothelial cells that lack connect to a stromal vessel. **(G–J)** are oriented with antrum at the top, granulosa cells central, and stroma at the bottom of each image/model; **(G,I)** at the same magnification.

Control IgG-injected follicles were well-luteinized, with an enlarged granulosa cell layer containing hypertrophied granulosa cells (Figures 6A,C). In contrast, the granulosa cell layer of THBS1 antibody-injected follicles was significantly thinner (Figures 6B,D). Granulosa cells appeared smaller and more densely packed when compared with control IgG-injected follicles (compare Figure 6A with Figure 6B; compare Figure 6G with Figure 6I). Where an expanded granulosa cell layer was noted in THBS1 antibody-injected follicles, the cells had foamy-appearing cytoplasm, which contrasted with the smooth appearance of granulosa cell cytoplasm in control IgG-injected follicles. THBS1 antibody-injected follicles showed more evidence of granulosa cell expansion in the granulosa cells close to the follicle antrum, while granulosa cells along the basement membrane remained more densely packed.

Follicular angiogenesis was evident in both control IgG-injected and THBS1 antibody-injected follicles. Vascular endothelial cells are restricted to the follicle stroma before hCG or the ovulatory LH surge (3). In the present study, staining for VWF identified vascular endothelial cells in the granulosa cell layer of both control IgG-injected and THBS1 antibody-injected follicles obtained 48 h after hCG (Figures 6A,B). In control IgG-injected follicles, VWF+ cells extended near to the antral edge of the luteinizing granulosa cells (Figures 6A,E), penetrating 89 ± 5% of the granulosa cell layer (Figure 6F). 3D modeling shows that VWF+ cells within the granulosa cell layer formed capillary-like networks which initiate at larger stromal vessels and produced a multi-branched network of VWF+ cells within the granulosa cell layer (Figures 6G,H). Red blood cells were visible within VWF+ cell-bordered spaces (Figure 6A), highlighting the luminal spaces forming within this capillary-like network.

Antibody neutralization of THBS1 reduced angiogenesis in the primate ovulatory follicle. VWF+ cells were located predominantly near the granulosa cell basement membrane of THBS1 antibody-injected follicles (Figure 6B). VWF+ cells penetrated less of the granulosa cell layer (Figure 6E) when compared to control IgG-injected follicles, reaching only 32 ± 7% of the distance between the granulosa cell basement membrane and the antral edge of the expanding granulosa cell layer (Figure 6F). 3D modeling of a THBS1 antibody-injected follicle confirms that VWF+ cells are present among granulosa cells, but groups of VWF+ cells do not connect back to stromal vessels (Figures 6I,J). In addition, less branching of capillary-like structures was seen in THBS1 antibody-injected follicles when compared with control IgG-injected follicles (Figure 6J). Capillary luminal spaces containing red blood cells were not observed in the THBS1 antibody-injected follicles (Figure 6B). However, the antrums of THBS1 antibody-injected follicles contained more numerous red blood cells (Figures 5D,H,I) when compared with control IgG-injected follicles (Figure 5G).

## Discussion

Angiogenesis is an essential component of the ovulatory cascade, since follicular angiogenesis is required for follicle rupture and oocyte release [reviewed in (1)]. Formation of new capillaries within the ovulatory follicle is initiated by the ovulatory LH surge. On a cellular level, angiogenesis is regulated by the coordinated effects of many vascular growth factors. In the primate ovulatory follicle, increased permeability of stromal vessels provides components for follicular fluid and permits immune cell movement from the circulation into the follicle. Vascular remodeling requires both stability and flexibility; stromal vessels must remain intact while new capillaries form and branch into the luteinizing granulosa cell layer. The LH surge has previously been shown to regulate follicular expression of both pro-angiogenic and anti-angiogenic growth factors. In this report, we identify THBS1 as an LH-stimulated vascular growth factor necessary for ovulatory angiogenesis, follicle rupture, and oocyte release in primates.

The LH surge stimulates expression of thrombospondin family members by granulosa cells of monkey ovulatory follicles. *THBS1* and *THBS4* mRNA levels increased by 24 h after administration of the ovulatory gonadotropin stimulus. However, only THBS1 protein levels were increased by 36 h, very near the time of ovulation in this macaque species. Levels of *THBS2* mRNA did not change across the ovulatory interval. Immunostaining showed that granulosa cells are likely the primary source of THBS1 and THBS4 proteins in the ovulatory follicle, with strong staining in the granulosa cell layer. Other cell types of the ovulatory follicle may also express THBS family members. Moderate staining in stromal structures, including walls of established stromal vessels, was also noted. Previous studies in marmosets, cows, and rodent species have identified granulosa cells as the primary source of THBS family members in ovarian follicles (9, 11, 14). Granulosa cell expression of *THBS1* and *THBS2* were reported to be highest in atretic follicles, ovulatory follicles after the gonadotropin surge, and in the large luteal cells of the developing corpus luteum (9, 11, 14, 15). Both FSH and LH have been shown to stimulate THBS family member expression by granulosa cells *in vitro* (9, 14). This pattern of LH-stimulated THBS family member expression is similar to our previous report of changing VEGF receptor ligands in the monkey ovulatory follicle. In this previous study, follicular VEGFA levels peaked soon after the LH surge while PGF levels peaked at the end of the ovulatory period, just before ovulation (18).

Prostaglandins have been suggested as key regulators of ovarian thrombospondin expression. LH-stimulated elevation of PGE2 and PGF2α levels in primate follicular fluid (17, 23) correlate with peak *THBS1* and *THBS4* mRNAs (Figure 1). In the present study, blockade of prostaglandin synthesis with celecoxib prevented the rise in *THBS1* and *THBS4* mRNA observed just before ovulation. However, THBS1 and THBS4 protein levels were not affected. PGE2 is a critical mediator of the ovulatory LH surge (24), and several PGE2 receptors are necessary for ovulation in primates (25). PGF2α increases THBS1 and THBS2 mRNAs in the bovine corpus luteum, where THBS1 plays a role in luteal regression (5). PGF2α receptors present in granulosa cells of the primate ovulatory follicle and young corpus luteum are not capable of signal transduction (26), so the PGF2α pathway is not available to regulate expression of thrombospondins, despite elevated follicular PGF2α levels. PGF2α receptors in primate luteal cells can mediate PGF2α signals later in the luteal phase (26). While prostaglandins are not a key regulators of thrombospondin protein levels in the primate ovulatory follicle, prostaglandins may regulate thrombospondin expression and activity in the primate corpus luteum, especially during luteal regression.

THBS1 is a potent stimulus for follicular angiogenesis *in vitro* and *in vivo*. THBS1 increased monkey ovarian endothelial cell migration and capillary sprout formation *in vitro*. THBS1 only modestly increased endothelial cell proliferation and overall length of capillary sprouts formed *in vitro*. These findings are consistent with a role for THBS1 to promote the differentiation of an endothelial cell into a tip cell, which migrates away from an established vessel and leads the formation of a new capillary sprout (21). THBS1 did not appear to promote the formation of stalk cells, which proliferate and form the tube of the capillary and connects the migrating tip cell to the parent vessel to establish blood flow (21). In the monkey ovulatory follicle *in vivo*, endothelial cells formed branching capillary-like networks that extended from stromal vessels to the antral edge of the luteinizing granulosa cell layer. In contrast, antibody neutralization of THBS1 resulted in limited follicular angiogenesis, even though established stimuli of follicular angiogenesis [such as VEGFA, placental growth factor (PGF), and PGE2 (3, 18, 25, 27, 28)] were likely still present. In THBS1 antibody-injected follicles, endothelial cells showed limited penetration into the granulosa cell layer, with little or no branching of capillary networks. In addition, endothelial cells present in the granulosa cell layer failed to maintain connectivity to nearby stromal vessels. A similar phenotype was seen in a previous study where a follicular administration of a PGF antibody altered follicular angiogenesis and blocked ovulation in primates (18). In branching angiogenesis, the migrating tip cell instructs adjacent endothelial cells to form stalk cells through signaling systems such as the NOTCH pathway (29–31). Failure of stalk cells to form in THBS1 antibody-injected follicles suggests that THBS1 may be involved in communication between tip and stalk cells or for the establishment of the stalk cell phenotype.

THBS1 blockade led to extensive accumulation of red blood cells (RBCs) within the follicle antrum. THBS1 interacts with components of the extracellular matrix (ECM) to support the structural integrity of the ECM and is most often described as an inhibitor of protease activity (32–34). THBS1 neutralization by antibody injection may lead to excessive proteolysis of ECM components, degradation of cell-cell and cell-matrix adhesion proteins, and liberation of matrix-associated growth factors in the stroma surrounding ovulatory follicles. THBS1 neutralization may contribute to the degradation of junctional complexes, creating leaky vessels and thereby increasing vascular permeability (35, 36). For example, THBS1 interacts with integrins and collagen cross-linking sites to mediate cell adhesion and firmly attach endothelial cells to the surrounding matrix; THBS1 also stimulates the phosphorylation of adherens, which form junctions between adjacent endothelial cells (22, 37). Many vascular growth factors, including VEGFA, are associated with ECM; proteolysis liberates these growth factors to establish the gradient that guides tip cells during angiogenesis (38). Without inhibition from THBS1, proteases may excessively liberate growth factors, disrupting gradients that simultaneously promote angiogenesis and create stable vessels (39). Lastly, THBS1 is known to facilitate RBC adhesion to endothelial cells (34, 40). Our 3D model shows that THBS1 is needed for the formation of capillary stalks. In the absence of stalk formation and without THBS1 to promote adhesion of RBCs to endothelial cells, fenestrations in the stromal vessel required for sprouting angiogenesis may provide an opening through which RBCs can flow uncontrolled, contributing to the pooling of RBCs in the follicle antrum (38). The specific mechanism is unknown and will require further investigation. However, through one or perhaps multiple mechanisms, THBS1 may be a key regulator of vascular permeability in the primate ovulatory follicle.

Overall, our studies demonstrate that THBS1 acts as a pro-angiogenic factor as the primate preovulatory follicle transforms into a corpus luteum. Most published reports describe THBS1 as an anti-angiogenic factor [reviewed in (41)]. However, THBS1 can act as a pro-angiogenic factor to promote endothelial cell proliferation, migration, and overall vascular growth (7, 8, 42, 43). Interestingly, pro-angiogenic actions of THBS1 are seen primarily in developing systems, while anti-angiogenic actions of THBS1 are often associated with tumor growth and wound healing (41). Pro-angiogenic actions of THBS1 are typically mediated via the N-terminus region of the molecule, which can interact with cell surface receptors including LRP1 and SDC4 (44). In contrast, anti-angiogenic actions of THBS1 are often mediated via THBS1's type I repeats interacting with CD36 receptors or through sequestration of pro-angiogenic factors such as VEGFA (44). Expression of CD36 in the ovary corresponds with THBS1's established role as an inhibitor of angiogenesis during follicle development, contributing to follicle atresia (14). There is limited information regarding expression of THBS1 receptors in non-human primate or human ovulatory follicles. However, increased CD36 was associated with poor human granulosa cell function (45), while elevated expression of SDC4 in human cumulus cells correlated with improved outcomes for IVF patients (46). The ability of THBS1 to act as a pro-angiogenic or anti-angiogenic factor may also be determined by THBS1 concentration (47), again highlighting the complexity of this system. In the primate ovulatory follicle, pro-angiogenic actions of THBS1 clearly dominate.

THBS1 neutralization *in vivo* significantly compromised ovulation. Injection of the THBS1 antibody resulted in failure of follicle rupture in two of four cases, with two ovaries forming very small rupture sites. Well-controlled proteolysis is essential for rupture (1). Since THBS1 has been reported to inhibit breakdown of many of the connective tissue components present in the follicle wall and surrounding ovarian stroma (32–34), it is somewhat surprising that THBS1 neutralization did not lead to enlarged or multiple ruptures sites. Previous studies support a critical role for ovulatory angiogenesis in ovulatory events including follicle rupture (18, 25, 48, 49), so the positive impact of THBS1 on follicle rupture at ovulation may be secondary to THBS1's pro-angiogenic actions in the follicle. An additional interesting observation is that two of three oocytes identified associated with THBS1-antibody injected follicles did not resume meiosis. Histological observation of the few oocytes available in the present study suggests that at least some degree of cumulus expansion occurred during THBS1 neutralization. Cumulus expansion has been described as a necessary prerequisite for resumption of oocyte meiosis (50). However, additional pathways promoting oocyte meiotic progression may be independent of cumulus expansion (51, 52). While much remains to be learned, our studies point to a critical role for thrombospondins, and in particular THBS1, as a paracrine regulator of follicular angiogenesis and ovulation in primates. A more complete understanding of THBS1 actions may ultimately enhance treatments for infertility and point to new targets for contraceptive development.

## Data Availability Statement

All datasets for this study are included in the manuscript/Supplementary Files.

## Ethics Statement

Whole ovaries and ovarian biopsies were obtained from adult female cynomolgus macaques (Macaca fascicularis) at Eastern Virginia Medical School (Norfolk, VA). All animal protocols were conducted in accordance with the National Institutes of Health's Guide for the Care and Use of Laboratory Animals and were approved by the Eastern Virginia Medical School Animal Care and Use Committee.

## Author Contributions

HB, GC, PA, AM, and DD each contributed data to this manuscript, was involved in preparation of the manuscript, and approved the final version of this manuscript. DD obtained funding and performed statistical analysis.

### Conflict of Interest

The authors declare that the research was conducted in the absence of any commercial or financial relationships that could be construed as a potential conflict of interest.
