# Supplementary material for: Thrombospondin 1 (THBS1) Promotes Follicular Angiogenesis, Luteinization, and Ovulation in Primates
Source: Front Endocrinol (Lausanne). 2019 Nov 7;10:727. doi: 10.3389/fendo.2019.00727 (PMC6855263; doi:10.3389/fendo.2019.00727)
Supplement: Supplementary file 2 [file Table_2.docx]

Supplemental Table 2. Primers for qPCR.

| Target | Primer Sequence (5’-3’) | Accession Number | Species |
| --- | --- | --- | --- |
| ACTB | Up ATCCGCAAAGACCTGT  Down GTCCGCTAGAAGCAT | NM_001285025.1 | Macaca fascicularis |
| THBS1 | Up GGTGACGTGACAGAAAAC  Down GCACTTCTTTGCACTCAT | XM_005559147.2 | Macaca fascicularis |
| THBS2 | Up TCAGACGGCCAACTCG  Down CCGGAGTAGCCATAGG | XM_005551410.2 | Macaca fascicularis |
| THBS4 | Up AGCTTCTACGTGGTCA  Down GCTTCATAAAATCGTACCC | XM_015140333.1 | Macaca mulatta |
